# The PPN and motor control: Preclinical studies to deep brain stimulation for Parkinson’s disease

**DOI:** 10.3389/fncir.2023.1095441

**Published:** 2023-02-28

**Authors:** Caixia Lin, Margreet C. Ridder, Pankaj Sah

**Affiliations:** ^1^Queensland Brain Institute, University of Queensland, St Lucia, QLD, Australia; ^2^Joint Centre for Neuroscience and Neural Engineering, and Department of Biology, Southern University of Science and Technology, Shenzhen, Guangdong Province, China

**Keywords:** motor control, basal ganglia, cholinergic, movement, gait

## Abstract

The pedunculopontine nucleus (PPN) is the major part of the mesencephalic locomotor region, involved in the control of gait and locomotion. The PPN contains glutamatergic, cholinergic, and GABAergic neurons that all make local connections, but also have long-range ascending and descending connections. While initially thought of as a region only involved in gait and locomotion, recent evidence is showing that this structure also participates in decision-making to initiate movement. Clinically, the PPN has been used as a target for deep brain stimulation to manage freezing of gait in late Parkinson’s disease. In this review, we will discuss current thinking on the role of the PPN in locomotor control. We will focus on the cytoarchitecture and functional connectivity of the PPN in relationship to motor control.

## Introduction

In mammals, coordinated movement of the limbs mimicking walking or running can be achieved without the cortex. This was first demonstrated in decerebrated cats where only the brainstem and cerebellum remained intact. In these animals, electrical or chemical stimulation of neurons in so-called brain locomotor regions produces locomotion. The mesencephalic locomotor region (MLR), consisting of the pedunculopontine nucleus (PPN) and adjacent cuneiform nucleus (CnF) was the first identified locomotor region and is present in all classes of vertebrates. Stimulation of this region in decerebrated cats evoked walking, trotting, or even galloping, depending on the stimulation strength (Shik et al., 1966a,b). A myriad of studies confirmed these initial observations and as a result, the MLR, and its projections, have primarily been thought to be involved in the control of movement. In contrast, goal-directed voluntary movements, such as stepping out of an elevator are initiated by and require cortical structures. However, recent results suggest that the PPN may also be involved in goal directed voluntary movement, suggesting it is also involved in the decision to move (Inagaki et al., 2022). The PPN’s involvement in both the automatic process of gait and goal-directed gait is perhaps not surprising as the numerous putative PPN connections, which have mostly been studied in rodents, contain descending as well as ascending projections to a variety of motor-related areas (Gut and Winn, 2016).

The involvement of the basal ganglia (BG) in the production of movement is evident in Parkinson’s disease (PD). While PD is a somewhat broad disorder with both motor and non-motor symptomatology (Schulz et al., 2011; Kalia and Lang, 2015; Poewe et al., 2017), the cardinal symptoms are motor and characterized by akinesia, tremor and gait abnormalities (Schulz et al., 2011; Kalia and Lang, 2015; Poewe et al., 2017). These motor deficits are thought to be due to the loss of dopaminergic neurons in the midbrain, most prominently the substantia nigra pars compacta (SNc), and a resultant reduction in dopamine in the basal ganglia. As such, to date, dopamine replacement, traditionally with L-DOPA (levodopa) or other dopamine agonists remains the standard treatment for the motor symptoms of PD. Unfortunately, dopamine replacement is often ineffective in patients with advanced PD symptoms such as freezing of gait (FOG) and postural instability (Giladi, 2008). FOG is an intermittent failure to initiate or maintain walking and is one of the most common reasons for patients to fall (King et al., 2020). The pathophysiology of FOG remains poorly understood but is associated with deficits in cognitive function and goal-directed motor planning (Knobl et al., 2012). Notably, cognitive functional impairments due to damage to the cerebral cortex, BG, or cerebellum can also disturb posture-gait control and result in falling.

In patients with advanced FOG, problems with the initiation of movement and falls lead to a significant loss in their quality of life. Dopamine replacement is not very effective for FOG and deep brain stimulation (DBS) of the PPN has emerged as a treatment for FOG relief for some patients (Mestre et al., 2016). Early research suggested that cholinergic neurons in the PPN were the key components for locomotor control (Garcia-Rill and Skinner, 1987), and post-mortem PD tissue studies found significant loss of cholinergic cells in the PPN (Hirsch et al., 1987; Zweig et al., 1989). However, using more selective stimulation strategies, this view has been challenged and instead suggested a larger role for glutamatergic neurons in both the PPN and CnF (Takakusaki et al., 2003; Sherman et al., 2015; Roseberry et al., 2016). Neither the CnF nor the PPN have clear anatomical boundaries and the precise location of the locomotor regulation region remains a matter of debate (Yelnik, 2007; Zrinzo et al., 2007; Thevathasan et al., 2012). Moreover, the mechanism by which DBS of the PPN relieves FOG also remains unknown. Due to a lack of knowledge about both the anatomical structure of the MLR as well as the circuity mechanism of FOG relief, it is not surprising that results have been variable (Thevathasan et al., 2018). This review will focus on the functional connectivity of the PPN in relationship to motor control, largely obtained from studies in rodents, how this may help understand human motor circuits, and perhaps develop better treatment options for movement disorders.

## Anatomy and cellular diversity of the PPN

The PPN is the major component of the MLR located in the caudal mesencephalic tegmentum. The anatomical and overall morphological structure of the PPN appears similar in all vertebrates. However, the exact boundaries that define PPN in humans are still not clear (Windels et al., 2015). The PPN is bounded laterally by the medial lemniscus, and medially by the superior cerebellar peduncle and its decussation. Caudal to the PPN is the retrorubral field and rostrally it is adjacent to the posterolateral substantia nigra. It is bounded caudally on its dorsal portion by the CnF and ventrally by the pontine reticular formation (Pahapill and Lozano, 2000; Jenkinson et al., 2009).

The PPN has a complex cytochemical architecture, formed by populations of cholinergic, glutamatergic, and gamma-aminobutyric acid (GABA)ergic neurons (Alam et al., 2011). Based on cytoarchitecture and neurochemical markers, it was initially subdivided into the caudal pars compacta (PPNc), consisting of a cluster of large neurons and the more rostral pars dissipata (PPNd; Mesulam et al., 1983; Geula et al., 1993; Pienaar et al., 2017), a nomenclature that has largely fallen out of favor. However, cholinergic and glutamatergic neurons are more abundant in caudal regions, while GABAergic neurons do not follow the same gradient, being more abundant in the rostral PPN (Pienaar et al., 2017). Some studies have suggested that choline acetyltransferase (ChAT) and GABA are colocalized in the somas and terminals of PPN neurons suggesting a dual release of acetylcholine and GABA (Jia et al., 2003). However, direct evidence for this is lacking and immunohistochemical findings indicate that PPN neurons are unlikely to have the co-release of acetylcholine with either glutamate or GABA as most cholinergic neurons in the PPN do not express the vesicular transporter for glutamate or enzymes for the synthesis of GABA (Wang and Morales, 2009).

## Anatomical and functional connectivity of the PPN

**Figure 1:**
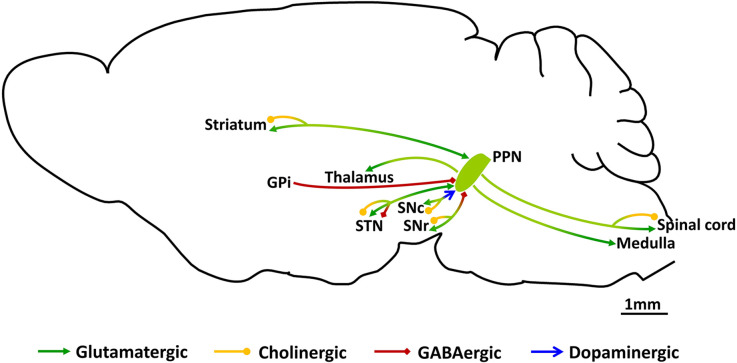
Schematic of synaptic connections of PPN neurons. PPN, pedunculopontine nucleus; GPi, the globus pallidus pars interna; SNr, substantia nigra pars reticulate; SNc, substantia nigra pars compacta; STN, subthalamic nucleus.

The PPN, acting as a transit station in locomotor control, receives motor commands from the upstream motor areas and in turn, sends ascending as well as descending projections to motor areas (Goulding, 2009; Figure 1). Synaptic input to the PPN arises from several motor-related regions with the strongest input from the BG (Goulding, 2009; Mori et al., 2016; Caggiano et al., 2018; Tubert et al., 2019; Dautan et al., 2021). The largest input is GABAergic arising from the substantia nigra pars reticulate (SNr) and the internal globus pallidus (GPi; Shink et al., 1997; Takakusaki et al., 2003, 2004). The PPN also receives glutamatergic input from the subthalamic nucleus (STN; Jackson and Crossman, 1981), and dopaminergic input from the substantia nigra pars compacta (SNc; Ryczko et al., 2016). Input from the SNr has been reported to inhibit PPN neurons targeting both the soma and dendrites (Granata and Kitai, 1991), but the exact targets, or physiological impact of GPi or STN input are not clear.

As previously mentioned, the PPN was initially defined by the distribution of large cholinergic neurons, and early studies on projections from the PPN focused on cholinergic cells (Garcia-Rill et al., 2019). These neurons are largely present in the caudal PPN, and send projections to the SNc, SNr and STN, with the largest projection to the SNc. Activation of this input to dopaminergic neurons in the SNc drives an inward depolarizing current mediated largely by nicotinic acetylcholine receptors (Futami et al., 1995; Xiao et al., 2016) that drives neural activity (Xiao et al., 2016). The SNr also receives cholinergic afferents, but these appear to activate M4 muscarinic receptors located on axon terminals carrying D1 input from the striatum (Moehle et al., 2017). However, little is known about the physiological impact of cholinergic PPN input on the SNr or STN. Ascending glutamatergic afferents from the PPN target many of the same regions of the BG that project to the PPN with input to the SNc, SNr, STN and striatum (Bevan et al., 1995; Rohrbacher et al., 2000; Galtieri et al., 2017). In the SNc, these afferents target dopaminergic neurons, forming excitatory synapses (Galtieri et al., 2017). The SNr which mainly consists of GABAergic neurons receives a dense projection from the PPN (Rohrbacher et al., 2000), and stimulation of these afferents evokes excitatory postsynaptic potentials (EPSPs) which are partly blocked by glutamatergic antagonists suggesting that input to the SNr is partly glutamatergic. Tracing studies have shown that both glutamatergic and GABAergic axon terminals from the PPN innervate the STN (Bevan et al., 1995). Outside the BG, the PPN sends glutamatergic (Assous et al., 2019; Dautan et al., 2020) and cholinergic (Dautan et al., 2020) input to the striatum that innervates local interneurons. Finally, a recent study has reported the existence of a glutamatergic projection from the PPN to the motor thalamus (Inagaki et al., 2022) innervating parts of the ventral medial (VM), ventral anterolateral (VAL), mediodorsal (MD), and intralaminar (IL) nuclei.

Descending PPN projections, initially studied in the decerebrated cat (Shik et al., 1966a, b), strongly project to the lower brainstem and medulla (Martinez-Gonzalez et al., 2011). Moreover, anatomical tracing studies show the presence of both cholinergic and glutamatergic projections to the spinal cord (Spann and Grofova, 1989; Sherman et al., 2015).

## The role of the PPN in locomotor control

Together with the CnF, the PPN is part of the MLR and participates in a diverse array of functions. It is involved in posture and gait control, sleep-wake regulation, cognition, and learning (Saper et al., 2010; Mena-Segovia and Bolam, 2011; Petzold et al., 2015). Functionally, chemical activation of the dorsal MLR leads to the movement (locomotion), while activation of the ventral MLR induced stopping (Sherman et al., 2015). Consistent with these findings, stimulation in the dorsal PPN induced stepping movements of cat hind limbs, while stimulation of the ventral part of the PPN caused inhibition of muscle tone (Takakusaki et al., 2016). Furthermore, lesioning of the MLR, including the PPN, leads to cataplexy and episodic immobility of gait (Sherman et al., 2015). These studies suggest that different subparts of the PPN/MLR contribute to different aspects of locomotor control.

Glutamatergic and cholinergic neurons are the main excitatory projection neurons in the PPN/MLR and have been suggested to play different roles in locomotor regulation. However, a variety of contradictory results have been reported. Initial studies were focused on cholinergic neurons due to their strong involvement in PD, with loss of PPN cholinergic neurons reported in PD patients. This disruption was associated with the gait impairment and cognitive deficits seen in PD patients as well as in animal PD models (Perry et al., 1985; Zweig et al., 1989; Karachi et al., 2010; Bohnen and Albin, 2011; Müller and Bohnen, 2013; Perez-Lloret and Barrantes, 2016). Experimentally, cholinergic neurons were first described to be required for gait in rodents (Kucinski and Sarter, 2015; Xiao et al., 2016) and selective lesioning of cholinergic PPN neurons in monkeys induced gait and postural impairments (Karachi et al., 2010). However, another study reported that in rodents, neither nonspecific lesioning of the PPN, or selective lesioning of cholinergic PPN neurons induced gait abnormalities (Gut and Winn, 2015). More recently, advances in genetic techniques have contributed to better insight into the function of distinct PPN neuron populations in locomotion. However, the results from recent studies are still debated (Roseberry et al., 2016; Caggiano et al., 2018; Josset et al., 2018; Dautan et al., 2021), a summary of this data is detailed in Table 1.

**Table 1:** Summary of cell type-specific motor control of the PPN.

	**Activate**	**Silence**
MLR glutamatergic neurons	▪ **Stationary state**: **initiate locomotion** (Roseberry et al., 2016) ▪ **Running**: **robust locomotion** (Roseberry et al., 2016)	▪ **Inhibition** **of MLR glutamatergic Neurons impedes Running** (Roseberry et al., 2016)
MLR cholinergic neurons	▪ **Stationary state**: **No effect** (Roseberry et al., 2016) ▪ **Running: increase in speed** (Roseberry et al., 2016)	
MLR GABAergic neurons	▪ **Stationary state**: **No effect** (Roseberry et al., 2016) ▪ **Running: deceleration** (Roseberry et al., 2016)	
PPN glutamatergic neurons	➢ **Failed to initiate** (Josset et al., 2018) ➢ **Short photoactivation (10 ms) modifies locomotor pattern (muscles response; step cycle**; Josset et al., 2018) ➢ **Decreased locomotor speed** (Josset et al., 2018) • **Initiation (high-frequency stimulation >10 Hz, longer onset than activation of the CnF**; Caggiano et al., 2018) • **Ongoing: increase speed** (Caggiano et al., 2018) ˚ **Reduce motor activity** (Dautan et al., 2021)	➢ **Only long photoinhibition (1 s) of glutamatergic PPN stops *Locomotion*** (Josset et al., 2018) • **Bilateral silencing decreases speed** (Caggiano et al., 2018)
CnF glutamatergic neurons	➢ **Initiate locomotion (Long pulse: 10-ms pulse duration at 20 Hz for 1 s**; Josset et al., 2018) ➢ **Short activation (10 ms) modifies locomotor pattern (muscles response**; Josset et al., 2018) ➢ **Long activation (1 s) resets rhythm and induces running gaits** (Josset et al., 2018) • **Initiation speed** (Caggiano et al., 2018) ˚ **Increase motor activity** (Dautan et al., 2021)	➢ **Rarely stopped Locomotion** (Josset et al., 2018) • **Bilateral silencing decreases speed** (Caggiano et al., 2018)
PPN cholinergic neurons	➢ **Failed to initiate** (Josset et al., 2018) ➢ **Little effect on locomotor speed or gait** (Josset et al., 2018) • **Slow or stop on-going locomotion** (Caggiano et al., 2018)	➢ **Rarely stopped locomotion** (Josset et al., 2018)

Stimulation of glutamatergic neurons in the PPN has led to a variety of outcomes, with some studies reporting that activation stimulates or increases movement (Caggiano et al., 2018; Masini and Kiehn, 2022), while others report a reduction in movement (Dautan et al., 2021). This most likely results from targeting different glutamatergic neuronal populations, for example, descending projecting neurons involved in the automatic process of gait vs. the ascending projection neurons that may be involved in goal-directed voluntary movements. There does however appear to be an emerging consensus. Within the MLR, while stimulation of glutamatergic neurons of the CnF evokes rapid locomotor activity (Caggiano et al., 2018), stimulation of the PPN affects slower movements (Josset et al., 2018). What is becoming apparent is that within the PPN, glutamatergic neurons contain functionally diverse subgroups projecting to different brain regions. Thus, glutamatergic neurons that project to the SNr are involved in movement regulation as well as behaviors such as rearing and grooming while the spinal cord projecting glutamatergic neurons are related to body extension control (Ferreira-Pinto et al., 2021). Optogenetic activation of PPN glutamatergic input to the motor thalamus elicits cue-triggered motor initiation (Inagaki et al., 2022). Moreover, glutamatergic PPN neurons projecting to the BG show differences in both gene expression and location as compared to those projecting to the medulla and spinal cord (Ferreira-Pinto et al., 2021), again pointing to distinct populations.

With a strong role in locomotor control, not surprisingly, the PPN is affected in some movement disorders. Following on from its use as a target for treating PD, recent studies have begun targeting the PPN in animal models of PD. Thus, a very recent study reported that activating glutamatergic PPN neurons rescued locomotor function in PD mouse models (Masini and Kiehn, 2022). Applying a combination of chemogenetics and optogenetics, they found that selective activation of caudal glutamatergic PPN neurons contributed to the relief of motor deficits in PD mice, and these effects were independent of CnF neurons (Masini and Kiehn, 2022), suggesting that more attention may need to be drawn to the caudal part of the PPN in relation to the PPN and PD treatment. These recent rodent studies highlight the complex role of the PPN in locomotor control with involvement in movement control as well as motor initiation. Further studies are required to clarify the cell type specific contribution of PPN neurons to locomotion including their gene expression profiles and exact anatomical location to better understand their involvement in motor neural circuits. Better insight into PPN-related neural circuits will enable very specific circuit manipulation, helping improve DBS targeting as well as develop novel therapeutic interventions for movement disorders.

## Parkinson’s disease and the PPN

PD is the second most common neurological disorder with a global incidence of 17 per 100,000. PD is age-related, usually affecting adults over the age of 50, with the risk of developing PD being 1.5 times higher in males than in females (Beitz, 2014; Poewe et al., 2017). The pathological hallmark of PD is degeneration and loss of dopaminergic neurons in the SNc (Cuenca et al., 2019). The resulting loss of dopamine input to the striatum results in the cardinal symptoms of PD: bradykinesia, rigidity, tremor and postural imbalance. For the past 50 years, dopamine replacement therapy with levodopa has been and remains the mainstay pharmacological treatment for symptomatic relief of PD. The effectiveness of dopamine replacement therapy depends on different factors including age, disease stage and progression of symptoms (Ferreira et al., 2013). However, levodopa treatment is less effective as the disease progresses, and postural instability and gait difficulties increase (Park and Stacy, 2011; Jenner, 2015). On a cellular level, degeneration becomes apparent not only in the SNc but also in other brain regions including the PPN (Rinne et al., 2008; Hepp et al., 2013; Pienaar et al., 2013; Chambers et al., 2019). Late in PD, there is a loss of cholinergic neurons in the PPN (Rinne et al., 2008; Müller and Bohnen, 2013; Kucinski and Sarter, 2015), and ascending cholinergic fibers can have a role in motor control (Xiao et al., 2016). Thus, as with Alzheimer’s disease (Rabins and Lyketsos, 2006; Seltzer, 2006), delivery of anti-cholinesteres have been tried in PD. However, the results have been very variable and this therapy is not in common use (Chen et al., 2021).

When dopamine replacement therapy alone is no longer sufficient to relieve PD motor symptoms, DBS has become and is a still evolving treatment option. In DBS, electrodes are implanted into specific brain regions and an implanted stimulator provides frequency modulated electrical simulation resulting in therapeutic relief for motor symptoms (Benabid, 2003). For the past 20 years, the GPi and STN (Breit et al., 2001; Lozano et al., 2019) have been the DBS targets for PD that yield a marked improvement in motor symptoms. While the exact mechanism of action of DBS that provides therapeutic relief is not clear, there is evidence that stimulation in the STN or the GPi alters the oscillatory activity in the BG that is awry in PD (Guridi and Alegre, 2017). The parameters that determine the measurable effectiveness of DBS are the stimulation amplitude, frequency and pulse width as well as the stimulation paradigm. The optimal stimulation protocol varies from person to person and is often largely dependent on what works for an individual, as assessed by the neurologist. Any given location of the electrodes in the brain may contain a variety of cell types that are part of different neurocircuits and also may contain fibers of passage from distant brain regions that can be driven orthodromically or antidromically. There is consensus that there is room for improvement when it comes to manipulating neural networks to ameliorate movement disorders.

As PD disease progresses, many PD patients develop FOG (Zhang et al., 2021), described by patients as “having their feet glued to the floor”. As the body initiates forward movement but the feet remain in place, it is not surprising that FOG is associated with a high risk of falling and hospitalization with a substantial reduction in quality of life (Bloem et al., 2004). The inability to initiate a step often occurs when the on-going locomotor pattern requires adaptation (e.g walking around an object) and is exacerbated under time constraint (e.g., stepping out of an opening elevator door). The difficulty in self-initiating movement can sometimes be overcome by sensory cues like visual cues on the floor, with rehabilitation therapy taking advantage of sensory cues as a means to reduce FOG episodes (Ginis et al., 2018). Voluntary movements are often planned before being executed and not initiated until a sensory cue is presented. A possible explanation for why sensory cues may overcome FOG is that self-initiated and cue-triggered motor initiation may involve different parallel motor circuits in the brain.

Where FOG results from self-initiating circuit failure due to BG degeneration, the cue-triggered movement circuit bypasses the degenerated BG, using brain areas that are spared from degeneration to initiate movement. Where FOG results from self-initiating circuit failure due to BG degeneration, the cue-triggered movement circuit bypasses the BG, using brain areas that are spared from degeneration to initiate movement. If this is the case it is not surprising that dopamine replacement therapy and DBS of the GPi and STN are ineffective against FOG (Hausdorff et al., 2009; St George et al., 2010). Some studies reported that GPi-DBS and STN-DBS improved FOG during medicine-off periods, however, the outcomes are not satisfactory, especially in the medicine-on condition (Volkmann et al., 2004; Schlenstedt et al., 2017; Kim et al., 2019). Treatment-resistant gait disturbances like FOG promoted the investigation of alternative targets for DBS. The original interest in the PPN in relationship to PD began in the 1980s when neurodegeneration of cholinergic neurons in the PPN region was observed in late-stage PD. As the PPN receives strong efferent innervation from the BG, it was a potential target for DBS (Rinne et al., 2008; Pienaar et al., 2013; Chambers et al., 2019). The benefit of PPN DBS was shown in primate models of PD with low-frequency electrical stimulation (2–20 Hz) of the PPN relieving akinesia (Jenkinson et al., 2004, 2006). The first clinical reports showing the benefit of PPN DBS, found that bilateral PPN-DBS in PD patients without medication significantly improved gait and postural symptoms including FOG (Plaha and Gill, 2005; Stefani et al., 2007). Notably, unlike in primate models, all clinical studies used high-frequency (100–130 Hz) PPN stimulation.

Although the initial studies on the benefits of PPN-DBS on FOG have subsequently been confirmed (Wilcox et al., 2011), some have reported only a marginal benefit (Ferraye et al., 2010), and others showed no benefit at all(Wang et al., 2017; Yu et al., 2020). These discrepancies between groups are perhaps not surprising for a number of reasons. Firstly, programming of PPN DBS is made particularly challenging as FOG is not displayed readily like tremors, and benefits to FOG may not appear until days or weeks after electrode activation. Secondly, electrode placement varies between the reported studies as the targeting methods vary among groups. To date, there is no consensus as to the exact location where the electrode should be placed, with even the exact location of the PPN still being up for debate (Thevathasan et al., 2018; Tubert et al., 2019). Due to the unclear boundaries of this region, the PPN is more difficult to clearly identify using magnetic resonance imaging (MRI) of clinical field strengths (1.5T and 3.0T), compared with other DBS targets like the STN or GPi (Plantinga et al., 2014). Thus, the stereotactic placement of electrodes is more variable than for the STN or GPi (Zrinzo et al., 2008; Hamani et al., 2016). Although the recently developed 7T ultrahigh-field MRI provides higher-resolution neuroimages of the PPN (Cong et al., 2018; Wang et al., 2019), there are still limitations for clinical applications. Firstly, 7T scanners are not widely available for clinical use, the scanning is slow (Cong et al., 2018) and often not tolerated by some PD patients. Second, even if higher resolution MRI can be obtained, the PPN has no obvious fiber tracts or other anatomic features delineating its boundaries and the current boundaries of the PPN as depicted in current atlases seem somewhat arbitrary. Thus, stereotactic placement may be on the border or even just outside the presumed PPN. Thirdly, as discussed above, the PPN region does not have a homogenous cell population. Glutamatergic, GABAergic and cholinergic neurons are unevenly distributed throughout the PPN area with glutamatergic and cholinergic neurons projecting to a large variety of motor-related brain regions.

How stimulation within the PPN leads to therapeutic relief is not known. However, as PPN-DBS has been reported to improve both FOG as well as simple reaction tasks (Hirsch et al., 1987; Thevathasan et al., 2010; Fischer et al., 2015), it raises the possibility that PPN DBS is indeed acting on this cue-triggered movement initiation motor circuit with the ascending glutamatergic PPN neurons that feed into the corticothalamic motor planning loop (Inagaki et al., 2022). Advances in neurocircuit dissection using rodent models have progressed immensely in the last decade. The treatment of gait disturbance in PD patients with PPN DBS is ikely to yield inconsistent clinical outcomes until research groups and treating clinicians reach a consensus of the optimal targeting site in the PPN area, which may not be found until we identify and locate the neurons that are key in ameliorating FOG.

## Conclusions

Location and cell type specific neural activation studies in rodents have shown that the PPN plays a significant role in a variety of locomotion control circuits. Recent advances in circuit activation and visualization tools will help pinpoint the exact population of PPN neurons and the corresponding neural circuit related to these locomotion circuits. These studies are necessary to investigate the complex mechanisms that engage the PPN in locomotion modulation especially gait regulation, in order to unveil how DBS in the PPN relieves advanced PD gait symptoms. At a clinical level, due to the limitations of current techniques, it is not feasible to target a specific group of neurons or very specific locomotor circuits during traditional DBS surgeries. However, a better understanding of the functional diversity and movement circuits within the PPN by rodent studies will help improve PPN-DBS targeting for PD. By placing the electrode more caudally in the PPN, glutamatergic neurons would be preferentially stimulated. Furthermore, it seems that subpopulations of the PPN neurons projecting to different axonal targets displayed diverse distributions within the PPN, opening the door to circuit-specific manipulation as a treatment option for PD patients in the future. Undoubtedly, only when technologies for cell-type specific DBS become available, can they be utilized to improve clinical outcomes for PD patients.

## Author contributions

CL, MR, and PS: wrote the manuscript. All authors contributed to the article and approved the submitted version.
